# Forecasting daily attendances at an emergency department to aid resource planning

**DOI:** 10.1186/1471-227X-9-1

**Published:** 2009-01-29

**Authors:** Yan Sun, Bee Hoon Heng, Yian Tay Seow, Eillyne Seow

**Affiliations:** 1 Health Services & Outcomes Research, National Healthcare Group, Commonwealth Lane, Singapore; 2 Department of Emergency Medicine, Tan Tock Seng Hospital, Tan Tock Seng, Singapore

## Abstract

**Background:**

Accurate forecasting of emergency department (ED) attendances can be a valuable tool for micro and macro level planning.

**Methods:**

Data for analysis was the counts of daily patient attendances at the ED of an acute care regional general hospital from July 2005 to Mar 2008. Patients were stratified into three acuity categories; i.e. P1, P2 and P3, with P1 being the most acute and P3 being the least acute. The autoregressive integrated moving average (ARIMA) method was separately applied to each of the three acuity categories and total patient attendances. Independent variables included in the model were public holiday (yes or no), ambient air quality measured by pollution standard index (PSI), daily ambient average temperature and daily relative humidity. The seasonal components of weekly and yearly periodicities in the time series of daily attendances were also studied. Univariate analysis by t-tests and multivariate time series analysis were carried out in SPSS version 15.

**Results:**

By time series analyses, P1 attendances did not show any weekly or yearly periodicity and was only predicted by ambient air quality of PSI > 50. P2 and total attendances showed weekly periodicities, and were also significantly predicted by public holiday. P3 attendances were significantly correlated with day of the week, month of the year, public holiday, and ambient air quality of PSI > 50.

After applying the developed models to validate the forecast, the MAPE of prediction by the models were 16.8%, 6.7%, 8.6% and 4.8% for P1, P2, P3 and total attendances, respectively. The models were able to account for most of the significant autocorrelations present in the data.

**Conclusion:**

Time series analysis has been shown to provide a useful, readily available tool for predicting emergency department workload that can be used to plan staff roster and resource planning.

## Background

The ability to predict daily attendances at the emergency department (ED) of a hospital is valuable at a micro level for planning of staff rosters, and at a macro level for financial and strategic planning. Time series analysis has been applied in emergency medicine to forecast workload (patient volumes) and to study the impact of selected factors on the provision of patient care at ED [1-10]. A time series is a sequence of measurements made over time. If a forecasting method is used to predict the time series, the difference between the actual value and the predicted value measures the error in prediction. The ultimate test of any forecasting method is the size of these errors, and a best-fit model is a model which minimizes the error.

Most published studies using time series were based on seasonal factors only and were developed for forecasting overall demand for ED services [2-7]. Since there is wide variation in disease severity and acuity among patients presenting at the ED, clinical services and resources required will likewise vary considerably. The experiences gained from studies carried out in Western countries may not necessarily apply to local conditions, as there are multiple factors that might contribute to the fluctuation of the daily attendances at an ED in Singapore.

The purpose of this paper is to identify the local factors associated with the daily attendances at ED, and to make predictions based on these local factors. As resources are dependent on patient acuity levels, the forecast is also stratified by patient acuity categories (PAC).

## Methods

### Setting

The study was carried out in an emergency department in a major public sector acute care regional general hospital in Singapore. The hospital has the highest number of ED attendances and the highest proportion of acutely ill patients among five public sector acute care general hospitals in Singapore. Permission to conduct the study was granted by the Chairman, Medical Board of the hospital.

### Data

Data used in the study was counts of daily patient attendances at ED between July 2005 and March 2008 (1,005 days), extracted from the ED administrative database. Patients who presented at the ED were classified as P1, P2 and P3 by the patient acuity category scale (PACS) used in all public sector hospital emergency departments in Singapore for resource allocation. P1 cases are most acutely ill and need immediate clinical services and treatment, P2 being acutely ill but can wait to be treated, and P3 being the less acutely ill patients who can wait longer to receive services (Table 1). Other data collected for the study included public holiday, and local weather factors (ambient temperature, ambient air quality measured by PSI, and relative humidity). The selection of the potential predictors was based on literature, local observation and availability of data. Singapore is a tropical country where the range in daily temperature throughout the year does not vary very much, hence daily average temperature was used.

**Table 1:** Patient classification by patient acuity category*

Patient acuity category	Description
P1	Patients of resuscitation, cardiovascular collapse or imminent danger of collapse, required to be attended to without a moment's delay

P2	Patients of non-resuscitation, major emergency or ill and non-ambulant or having severe symptoms and trolley based

P3	Patients of minor emergency or ambulant with mild to moderate symptoms

* Definition given by Ministry of Health (MOH) of Singapore

### Study design and methods

Univariate analysis of daily ED attendances and their association with potential predictors was carried out using general linear model, and significance testing using t-test where probabilities > 0.05 was considered statistically significant.

Time series analysis for identifying significant predictors as well as for forecasting daily ED attendances were carried out using established time series analysis procedures, the most popular time series analysis technique being auto regression integrated moving average (ARIMA) [11] model. ARIMA is a class of models, which are represented by (*p*, *d*, *q*)(*P*, *D*, *Q*)_*S*_, where *p *is the order of autoregression, *d *is the order of differencing (or integration), and *q *is the order of moving-average; (*P*, *D*, *Q*) are their seasonal counterparts; and *s *is the seasonal period [12]. Both weekly and yearly seasonal periodicities were taken into account in this analysis.

ARIMA models were iteratively applied to P1, P2, P3 and total patient attendances using data of the first 24 months to train, data of the following 6 months to test, and that of the following 3 months to validate. Elsewhere, models are usually trained and their performance evaluated on the test data; finally the model with least error is chosen as best-fit model. This strategy, however, leads an optimistic estimation of the performance of the chosen model since the data used for training and testing are identical with the data used for performance evaluation. Therefore, in this study, we used a third data set for performance evaluation (model validation).

The model with the lowest mean absolute percentage error (MAPE) calculated on the test data and a non-significant Ljung-Box test (p ≥ 0.05) was chosen as the best-fit model, where MAPE was defined as [13]:

$$MAPE=\sum_{i=1}^{N}\frac{\left|{\tilde{x}}_{i}-x_{i}\right|}{x_{i}}$$

where *x*_i _denotes the observed number of daily attendances at date i, ${\tilde{x}}_{i}$ denotes the predicted value of *x*_i_. Ljung-Box test is commonly used in ARIMA model for measuring the difference between the real time series and predicted series by the model. A non-significant p-value (≥ 0.05) of the test means that the model well represents the observed time series. A MAPE of 0% denotes a perfect fit of the model when applied to the validation dataset. The best-fit model was then used to forecast prospectively and validated. As far as we know, there is no specific definition of "good accuracy" of a model. It is usually taken to be a non-significant p-value of the model by Ljung-Box test (p < 0.05) and a MAPE of < 20%. If the MAPE is less than 5%, the model performance can be regarded as being excellent.

Independent variables included in the model as potential predictors of daily ED attendances were public holiday (yes/no), ambient air quality measured by pollution standards index (PSI), average daily ambient temperature and average daily relative humidity. The seasonal components of weekly and yearly periodicities in the time series of daily attendances were also studied. The National Environmental Agency (NEA) of Singapore adopts the PSI developed by the US Environmental Protection Agency that provides easily understandable information about daily levels of air pollution. A range of 1–50 is considered good, while that 51–100 was moderately unhealthy, and >= 100 was unhealthy [14]. The readings on most days in Singapore were within good range. Therefore, we categorized PSI (> 50 and <= 50) for better statistical power.

The predictors at preceding days may also affect current ED attendance, or a lag association. It is defined as correlational dependency of order *k *between each *i*'th element of the series and the (*i-k*)'th element and measured by autocorrelation (i.e. a correlation between the two terms), and *k *being the lag [15].

All statistical analyses were done in SPSS version 15, using automated identification of best-fit models from each dependant variable based on performance measure, where probabilities less than 0.05 was considered statistically significant. Lag association was also automated by SPSS.

## Results

### Descriptive analysis

On an average, there were about 400 daily attendances at the ED during the period July 2005 to Dec 2007. These comprise 8% P1, or approximately 30 cases per day. P2 and P3 patients together accounted for about 92% of total daily attendances (Table 2). About 70% of P1 attendances were for severe respiratory and heart conditions; while approximately 80% of P3 attendances were for trauma, viral infection and gastrointestinal diseases. P2 cases were a combination of P1 and P3 dominant conditions. Significant daily variations were noted, with daily P1 attendances ranging from 10 to 72 cases, P2 attendances ranging from 96 to 239 cases, and P3 attendances ranging from 138 to 307 cases.

**Table 2:** Mean daily attendances at emergency department by patient acuity category

	Mean daily attendances (95% confidence interval)		
	
Patient acuity category	Training data	Testing data	Validation data
P1	30.1 (29.5–30.7)	31.6 (30.6–32.6)	32.8 (31.4–34.1)
P2	162.4 (160.8–164.1)	178.5 (175.8-11.3)	198.5 (194.6–202.4)
P3	204.5 (202.2–206.8)	211.4 (207.1–215.8)	201.0 (194.9–207.1)

All	400.4 (397.5–403.2)	425.1 (419.9–430.2)	435.4 (428.4–442.3)

P1: Patients of resuscitation, cardiovascular collapse or imminent danger of collapse, required to be attended to without a moment's delay

P2: Patients of non-resuscitation, major emergency or ill and non-ambulant or having severe symptoms and trolley based

P3: Patients of minor emergency or ambulant with mild to moderate symptoms

**Figure 1:**
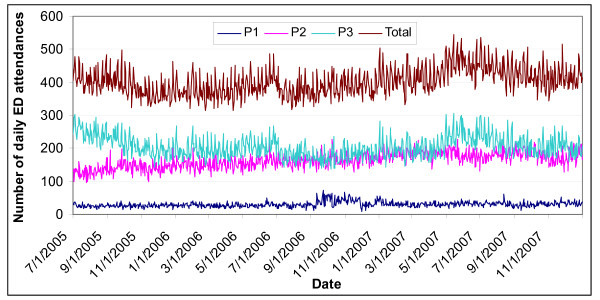
**Daily attendances at emergency department by patient acuity categories, Jul 2005 to Dec 2007**.

**Figure 2:**
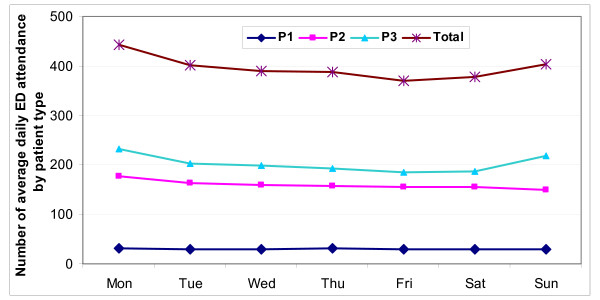
**Average daily attendances at emergency department by day of the week, Jul 2005 to Dec 2007**.

**Figure 3:**
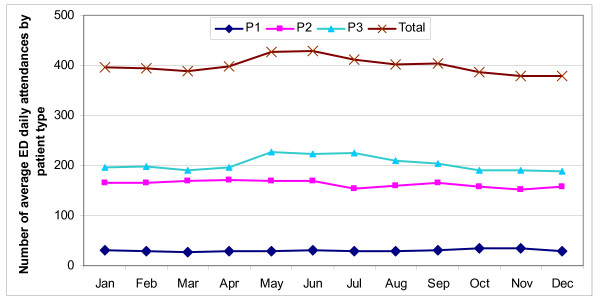
**Average daily attendances at emergency department by month of the year, Jul 2005 to Dec 2007**.

The secular trend is one of increasing trend in total attendances, especially from 2006 onwards (Fig. 1). Fig. 2 shows weekly fluctuations. The higher total attendances on Monday were contributed mainly by P2 and P3 cases, while higher attendances on Sunday were contributed by P3 cases. Fig. 3 shows higher attendances from May to July, being contributed mainly by P3 cases. There was no yearly fluctuation in P1 attendances.

### Univariate analysis

Table 3 shows a significant upward secular trend in the number of attendances; with a monthly increase of 2.2 total attendances during the study period. These were contributed by a monthly increase of 0.3 cases of P1 and 2.1 cases of P2. On public holidays, there was an average of 18 more P3 attendances per day. Average ambient temperature was associated with about 6 more P3 attendances per Celsius degree increase. Moderate ambient air quality (PSI > 50) was correlated with an average of 8–9 more P1 and P2 attendances per day. Overall, humidity was negatively correlated with P1 and P2 cases.

**Table 3:** Univariate analysis of daily attendances at emergency department by predictors

	P1		P2		P3		Total	
	
Predictors	MD	p value*	MD	p value	MD	p value	MD	p value
Time (in months)	0.3	< 0.001	2.1	< 0.001	-0.1	ns	2.2	< 0.001
Day of the week: Sun	0.9	ns	-4.6	ns	34.3	< 0.001	27.6	< 0.001
Mon	3.0	0.005	19.8	< 0.001	48.0	< 0.001	66.5	< 0.001
Tue	0.5	ns	8.0	0.005	17.6	< 0.001	26.0	< 0.001
Wed	1.1	ns	2.7	ns	11.7	0.001	10.6	0.014
Thu	1.4	ns	0.8	ns	9.3	0.008	10.3	0.016
Fri	0.3	ns	-1.3	ns	-1.1	ns	-6.8	ns
[Sat]	0.0	-	0.0	-	0.0	-	0.0	-

Month of the year: Jan	0.1	ns	0.1	ns	5.5	ns	5.3	ns
Feb	-2.2	ns	0.3	ns	6.7	ns	3.9	ns
Mar	-3.5	0.012	3.8	ns	-1.6	ns	-2.0	ns
Apr	-2.3	ns	5.4	ns	4.9	ns	7.8	ns
May	-2.1	ns	3.1	ns	35.2	< 0.001	37.0	< 0.001
Jun	0.4	ns	4.3	ns	31.4	< 0.001	37.9	< 0.001
Jul	-2.5	0.041	-10.7	0.002	33.6	< 0.001	21.0	< 0.001
Aug	-2.5	0.039	-6.3	ns	18.9	< 0.001	11.3	ns
Sep	-0.3	ns	0.2	ns	12.3	0.006	13.7	0.019
Oct	2.7	0.029	0.3	ns	1.3	ns	4.9	ns
Nov	3.2	0.011	-9.9	0.005	6.3	ns	-0.4	ns
[Dec]	0.0	-	0.0	-	0.0	-	0.0	-

Public holiday (Yes)	0.9	ns	-11.7	0.009	17.8	0.003	7.7	ns

Ambient temperature	0.0	ns	0.5	ns	5.5	< 0.001	6.2	< 0.001

Relative humidity	-0.1	0.039	-0.3	ns	0.3	ns	-0.8	0.007

PSI > 50 (Yes)	8.7	< 0.001	8.2	ns	-29.2	< 0.001	-13.2	ns

[]: reference group

PSI: pollution standards index

MD: mean difference

* using t-test

ns: not significant

### Time series analysis

As shown in Table 4, by Ljung-Box tests, the p-values of the best-fit models were not significant, which means all the four models closely represented the observed time series. The best-fit model for P1 was ARIMA(0,1,1), which is a non-seasonal and non-stationary moving average model. The best-fit model for P2 was ARIMA(1,1,1)(1,0,1), which is a seasonal non-stationary auto-regression integrated with moving average model. The best-fit models for P3 and total attendances were ARIMA(0,1,1)(1,0,1), which are seasonal non-stationary moving average model.

**Table 4:** Best-fit ARIMA models and their predictors by patient acuity category

				MAPE (%)	
				
Patient acuity category	Best-fit model	No. of predictors	Predictors (maximum lag correlation)	Test	Validation
P1	ARIMA(0,1,1)	1	PSI > 50 (2 days)	18.2	16.8
P2	ARIMA(1,1,1)(1,0,1)	1	Public holiday (1 day)	7.7	6.7
P3	ARIMA(0,1,1)(1,0,1)	2	Public holiday (1 day), PSI > 50 (0 day)	7.2	8.6

All	ARIMA(0,1,1)(1,0,1)	1	Public holiday (1 day)	4.4	4.8

ARIMA: auto-regression integrated moving average

MAPE: mean absolute percentage error

(p, d, q)(P, D, Q): p is the order of auto-regression, d is the order of differencing (integration), and q is the order of moving average; P, D, Q are their seasonal counterparts

All the four data series had linear trend since all 'd's in the best-fit models equal 1. P1 attendance did not show any weekly or yearly periodicity and was only predicted by ambient air quality of PSI > 50. P2 and total attendances showed weekly periodicities in the time series analyses, and were also significantly correlated with public holiday. P3 attendance was significantly correlated with day of the week, month of the year, public holiday, and ambient air quality of PSI > 50. The maximum lag between PSI > 50 and P1 cases was two days; there was no lag between PSI > 50 and P3 cases. The maximum lag between public holiday and P2, P3 and total cases was one day (Table 4).

P1 yielded a MAPE of 16.9% on validation; or forecasts of the model had an average error of 6 out of an average 33 attendances per day. The models for P2, P3 and total attendances performed better in the daily prediction of attendances, with a MAPE of 6.7%, 8.6% and 4.8%, respectively.

**Figure 4:**
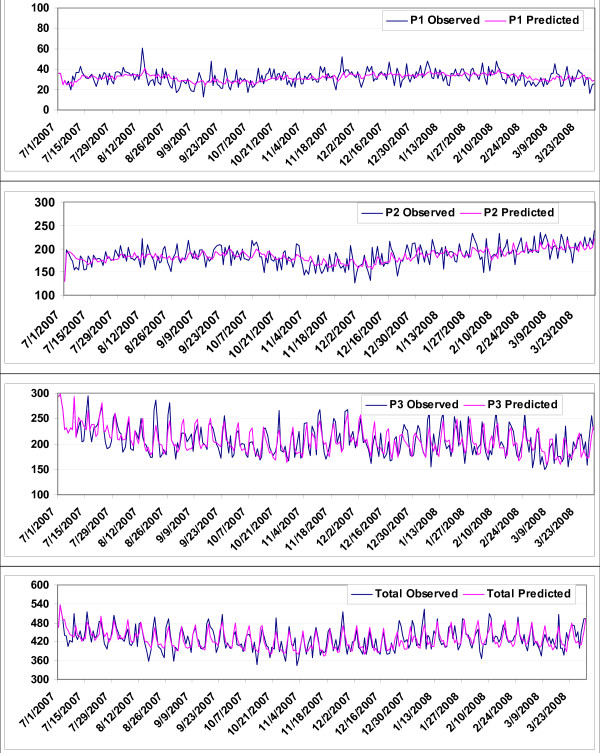
**Observed and predicted daily attendances at emergency department by patient acuity categories, Jul 2007–Mar 2008**.

**Figure 5:**
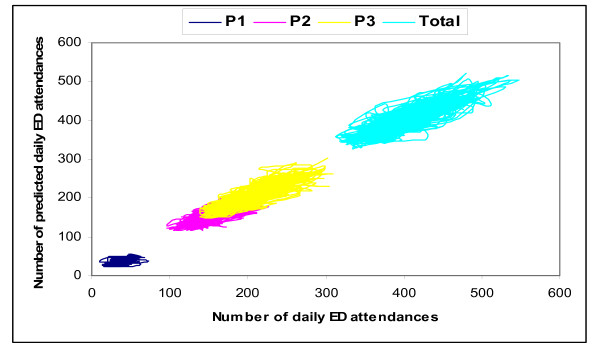
**Scatter plot of numbers of daily attendances at emergency department by patient acuity categories, observed vs predicted, Jul 2007 – Mar 2008**.

Fig. 4 shows the observed and predicted time series for P1, P2, P3 and total attendances overlap with each other to a great degree. The scatter plots of observed vs predicted attendances by the four best-fit models shows that the points to be distributed along the diagonal line (Fig. 5); i.e. the models were successful in accounting for most of the significant autocorrelations present in the data.

## Discussion

Although emergencies are difficult to foresee, this study demonstrated that daily patient attendances at ED can be predicted with good accuracy using the modeling techniques in time series analysis. During the study period, the daily variations noted were quite significant, with daily P1 attendances ranging from 10 to 72; P2 attendances ranging from 96 to 239; P3 attendances ranging from 138 to 307. The model developed has identified factors associated with these variations in a local setting; which in turn were used to forecast future workload. Although the P1 model showed the highest prediction error due to the very small number of daily P1 attendances, it still demonstrated good forecasting ability.

Unlike other studies [6,8], this study showed that daily total ED attendances were not predicted by weather conditions. This could be because Singapore is a tropical city with little variation in its hot and humid weather conditions throughout the year. While there was no seasonal fluctuation, higher P1 attendances was predicted by moderate or poor ambient air quality (PSI > 50). This could be due to severe respiratory and heart diseases among the vulnerable elderly population, which make up the 70% of P1 cases, and as reported by other studies [16-18]. On the other hand, PSI > 50 was significantly inversely correlated with P3 attendances; i.e. fewer P3 attendances on days with high PSI. Singapore's national advisory on days with moderate to poor PSI follow that of US EPA; to reduce outdoor activities especially among those with compromised heart and lung conditions. Reduced outdoor activities during days of bad PSI may possibly account for this as attendances for trauma associated with minor accidents also decreased.

There were predictable higher weekly attendances on Sundays and Mondays, contributed by P3 cases. This is attributed by the closure of primary care facilities, mainly of the public sector on Sundays and public holidays; and the build-up of demand on Mondays. Similarly public holidays were also strongly correlated with higher P3 attendances when the primary care facilities are closed. There were also higher monthly attendances from May to July, contributed by P3 cases. This is attributed to the perennial seasonal dengue outbreaks and mid-year influenza activity.

Similar modeling and predicting framework can be extended to time series analysis of different intervals, such as hourly, weekly, monthly or yearly, as well as for different disease groups. The model's performance is based on historical trends. It is imperative for the forecasts to be iterative and updated regularly as more data is available in order to improve the prediction performance. In this case, the model is updated 3-monthly and the framework has been put into practice, where the model is run weekly to forecast the workload the following week. The forecasts have been used by the ED management to plan its staff deployment on a weekly base.

In addition to the immediate weekly forecasts, the model has also been used to plan longer term ahead. The study has shown higher daily P3 attendances due to the seasonal dengue and influenza outbreaks mid-year. Moreover, there were also higher P1 and P3 attendances associated with high PSI readings caused by transboundary air pollution from the seasonal forest fires in neighboring countries. These secular annual forecasts help the department plan staff headcounts and budget allocation a year in advance.

The study has helped us understand the factors associated with variation of daily ED attendances in a local setting and develop a model to forecast the daily attendances. To our knowledge, it is the first such study in Singapore. This study suffers from a few limitations. One is that there may be other factors affecting the daily ED attendances, like the availability of other primary care facilities and their workload which may predict ED attendances. Another limitation is the use of average daily temperature. Although the temperature range throughout the day may not be wide, maximum and minimum temperature could be more useful as a predictor. Also, we did not evaluate alternate forms of the predictor variables (e.g., squared, cubed or other non-linear forms) in this study, which may give better prediction of ED attendances.

## Conclusion

Forecasting methods are useful in healthcare management. Accurate prediction of patient attendances will facilitate timely planning of staff deployment and allocation of resources within a department or a hospital. The hospital where the study was carried out is a regional hospital, with its catchment of patients geographically determined. The approach proposed and lessons learned from this experience may assist other four regional hospitals and their emergency departments to carry out their own analysis to aid planning and budgeting. Overall, it allows for a basis of macro-planning and allocation of budget by the Ministry of Health, which up to now is based on an average aggregated incremental percentage annual growth.

## Competing interests

The authors declare that they have no competing interests.

## Authors' contributions

SY designed the study, did the data analysis and wrote the first draft. BHH, ES and SYT conceived the study and made substantial contributions to the discussion of the results, and contributed to manuscript drafts. All authors have read and approved of the content of the final submitted manuscript.

All authors have access to all data in the study and they hold final responsibility for the decision to submit for publication.

## Pre-publication history

The pre-publication history for this paper can be accessed here:


